# Diffusion-Weighted MRI in the Evaluation of Early-Stage Breast Cancer Treated with a Short Preoperative Radiotherapy: Preliminary Results

**DOI:** 10.5334/jbsr.2815

**Published:** 2023-02-07

**Authors:** Nathalie Hottat, Jacques Jani, Dominique Badr, Mark De Ridder, André Nazac, Katherina Vanden Houte, Sophie Lecomte, Mieke Cannie

**Affiliations:** 1 University Hospital Brugmann, BE; 2 UZ Brussel, BE

**Keywords:** apparent diffusion coefficient, diffusion-weighted MRI, early-stage breast cancer, Ki-67 index, neoadjuvant radiotherapy

## Abstract

**Objective:**

To assess tumor response with diffusion-weighted MRI (DW-MRI) after a short preoperative radiotherapy in early-stage breast cancer (BCa).

**Materials and Methods:**

This was a prospective, single-center pilot study. 3T-MRI were performed before and after radiotherapy. The longest diameter (LD) and the apparent diffusion coefficient (ADC) value of a region of interest (ROI) of the tumors were recorded. Histopathology and immunohistochemistry, including the Ki-67 index of the core biopsy and of the surgical specimen, were the reference standards.

**Results:**

Nineteen patients with 22 early-stage BCa were included. The mean ROI ADC value was 1.093 ± 0.278 × 10^-3^ mm^2^/s before radiotherapy and 1.490 ± 0.429 × 10^-3^ mm^2^/s (p-value < 0.001) after radiotherapy. The Ki-67 index was 9.2 ± 9.1% at the percutaneous biopsy before radiotherapy and 4.9 ± 7.5% (p-value = 0.005) after radiotherapy at the surgical specimen. After neoadjuvant radiotherapy, a 4.7% decrease in LD and a 36.3% increase in ROI-ADC of the tumors were measured at MRI and a 46.7% decrease in Ki-67 index was observed at histology of the surgical specimen in comparison with the percutaneous core biopsy.

**Conclusion:**

In early-stage BCa, a significant increase in ROI-ADC at DWI and a significant decrease in Ki-67 index were observed after a short preoperative radiotherapy, suggesting early tumor response.

## Introduction

Currently, women with early-stage breast cancer (BCa) (stage cT1-2N0M0) are treated with breast-conserving surgery and sentinel node biopsy, followed by whole-breast irradiation with a boost targeted on the lumpectomy cavity [1]. A six-week interval after surgery is advisable before the start of radiotherapy. Formerly, whole-breast irradiation with boost was delivered in six to seven weeks [2]. Preoperative radiotherapy followed by immediate breast-sparing surgery is a new therapeutic approach under study in our institution with potential benefits, such as a more precise targeting of the tumor and a decrease in overall treatment time with positive effect on quality of life and reduction in health care costs [3]. This therapeutic management could also avoid delays in delivering radiotherapy in case of post-operative wound complications.

Magnetic resonance imaging (MRI) with dynamic contrast-enhanced (DCE) sequences is the best imaging technique for assessing BCa response after neoadjuvant treatment and for predicting pathologic response, with an accuracy of approximately 91% [4,5,6,7]. Diffusion-weighted imaging (DWI) measures the mobility of water molecules in tissues and provides information regarding the tissue cellularity. This technique has been shown to increase the diagnostic accuracy of DCE MRI with a high specificity for the detection of malignant lesions [8]. Tumor lysis, loss of cell membrane integrity, and increased extracellular space are associated with an increase in water diffusion illustrating the treatment response. In fact, apparent diffusion coefficient (ADC) value increase is observed for BCa patients after chemotherapy, with larger increases in pathologic responders compared with non-responders [9,10]. Diffusion-weighted imaging is widely used in the MRI monitoring of BCa under treatment [11,12,13,14,15].

Our purpose was to prospectively study DWI in the evaluation of tumor response after neoadjuvant preoperative radiotherapy in early BCa.

## Materials and Methods

### Study design and patient selection

**Figure 1:**
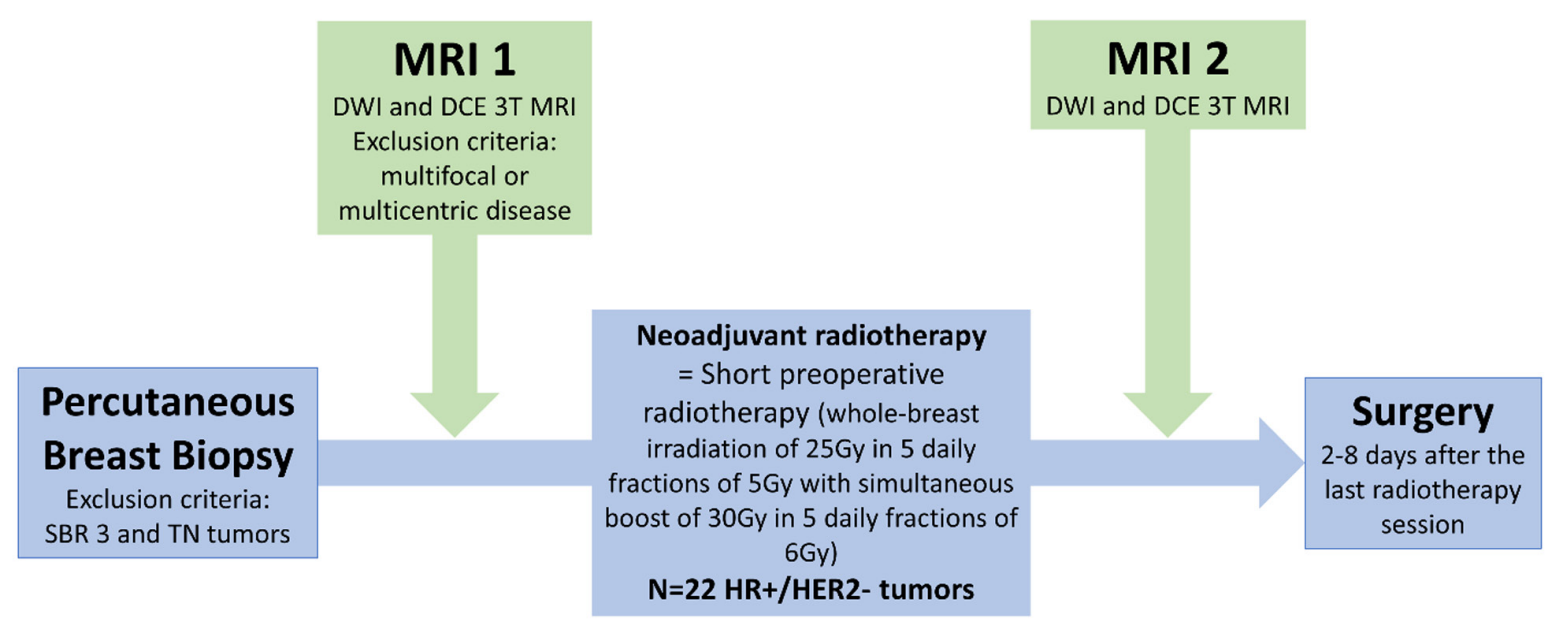
Flowchart of the study.

Between March 2017 and November 2020, DWI before and after preoperative radiotherapy was evaluated in a series of 19 patients with 22 early BCa enrolled in this prospective, single-center pilot study (ancillary study to the POPART study) [3]. The inclusion criteria were age over 18 years, a pretherapeutic standardized breast 3T MRI, stage cT1-2N0M0, Luminal A or B, candidate for breast-conserving surgery, N0-status confirmed by lymph node cytology, and a standardized breast 3T MRI after radiotherapy. The exclusion criteria were contraindication to MRI examination, multifocal or multicentric disease on pretherapeutic MRI, prior thoracic radiotherapy, pregnancy, Scarff-Bloom-Richardson (SBR) grade 3, and triple negative status. A short preoperative radiotherapy (whole-breast irradiation of 25 Gy in 5 daily fractions of 5 Gy with a simultaneous integrated boost of 30 Gy in 5 daily fractions of 6 Gy) was applied before surgery. Standardized MRI, including DWI and DCE sequences, was performed before and after radiotherapy. Subsequent conservative surgery with sentinel node biopsy was performed two to eight days after the last radiotherapy session and after the second standardized breast 3T MRI (Figure 1). This study was approved by the institutional Ethics Committee, and all patients signed informed consent forms. It was also registered in ClinicalTrials.gov (NCT02916719).

### Image acquisition

MRI examinations were performed in the prone position with a 3-Tesla system (Philips Ingenia, Best, Netherlands) using a 7-element SENSE breast coil. Gadoteric acid (0.2 mmol/kg; Dotarem 0.5 mmol/mL, Guerbet, Aulney, France) was injected with a power injector (Medrad, Maastricht, Netherlands) at a rate of 2 mL/sec, followed by 20 mL of saline solution. The MR imaging protocol included the following sequences: T2 SPAIR TSE, T1 TSE, diffusion-weighted single-shot EPI (b = 0, 50, 400, and 800 s/mm^2^) and dynamic eTHRIVE (3D T1-weighted gradient echo with fat saturation) following gadolinium administration, all acquired in the transversal plane (supplemental table 1). All images were sent to a PACS workstation.

### Image analysis

One radiologist with 15 years of experience in the field of breast MRI read the MRI examinations according to the BI-RADS Classification [16]. The DCE images were analyzed with the post-processing MR BREVIS tool of Syngo.via (Siemens Healthineers, Erlangen, Germany), and time intensity curves (TIC) were acquired in the most enhancing part of the tumor. Diffusion-weighed images were automatically co-registered with DCE images. Tumors were identified on DCE subtraction images and localized on DW images. A small circular two-dimensional (2D) region of interest (ROI) was placed on the darkest part of the lesion on the ADC map focused on the targeted restrictive area corresponding with the highest signal intensity on the highest b-value. The size of the ROI was adapted to the size of the restrictive tumoral target avoiding the surrounding fat or normal tissue and clip artefacts. For each patient, the same size of ROI was used before and after radiotherapy. In the absence of a targeted restrictive residual tumor, the ROI was placed in the same tissue region as in the previous examination. The longest diameter (LD) on DCE, the TIC, and the ROI ADC value of the tumor were recorded prospectively.

### Histopathology

The histopathology of the percutaneous core biopsy before radiotherapy and of the surgical specimen after radiotherapy were considered as the reference standards. The histological grading was determined according to the modified Scarff-Bloom-Richardson scoring and combined Nottingham classification. Tumors were classified based on immunohistochemistry (IHC) results according to the following molecular subtypes: Luminal A (estrogen receptor (ER)-positive, Ki-67 <20%, and human epidermal growth factor receptor 2 (HER2)-negative); Luminal B (ER-positive with either Ki-67 ≥20% or HER2-positive); HER2 enriched (ER-negative and HER2-positive); and triple-negative (ER-negative, PR-negative, and HER2-negative). The Ki-67 proliferation index was determined on IHC on the core biopsy and on the surgical specimen (with a Dako MIB-1 antibody). Pathologic CR (pCR) was defined as the absence of residual tumor in the breast and axilla (ypT0 and ypN0), independently of the presence of ductal carcinoma in situ.

### Statistical analysis

Data were analyzed with the SPSS 26 statistical software (IBM SPSS statistics) and R version 4.1.2. Continuous variables were expressed as mean ±1 standard deviation (SD), while categorical variables were expressed as number (frequency). The Shapiro-Wilk test was used to check the normal distribution of continuous variables. Either the t-test for related samples or the Wilcoxon test was used for comparison of means of paired samples, while McNemar’s test was used for comparison of paired proportions [17,18]. A p-value <0.05 was considered statistically significant.

## Results

Nineteen patients (median age 61 years, range: 48–79) were recruited and underwent standardized 3T breast MRI before and after preoperative radiotherapy. Among them, 18 women were in menopause. One menopausal woman took a hormone replacement therapy stopped two weeks before the first MRI. Three patients had a bilateral BCa. Supplemental table 2 presents the characteristics of the 22 tumors: 18 were no special type carcinomas and 4 invasive lobular carcinomas, among them 13 were SBR 1 and 9 SBR 2, all had a HR-positive/HER2-negative IHC profile, and 18 were Luminal A and 4 Luminal B at percutaneous biopsies.

**Figure 2:**
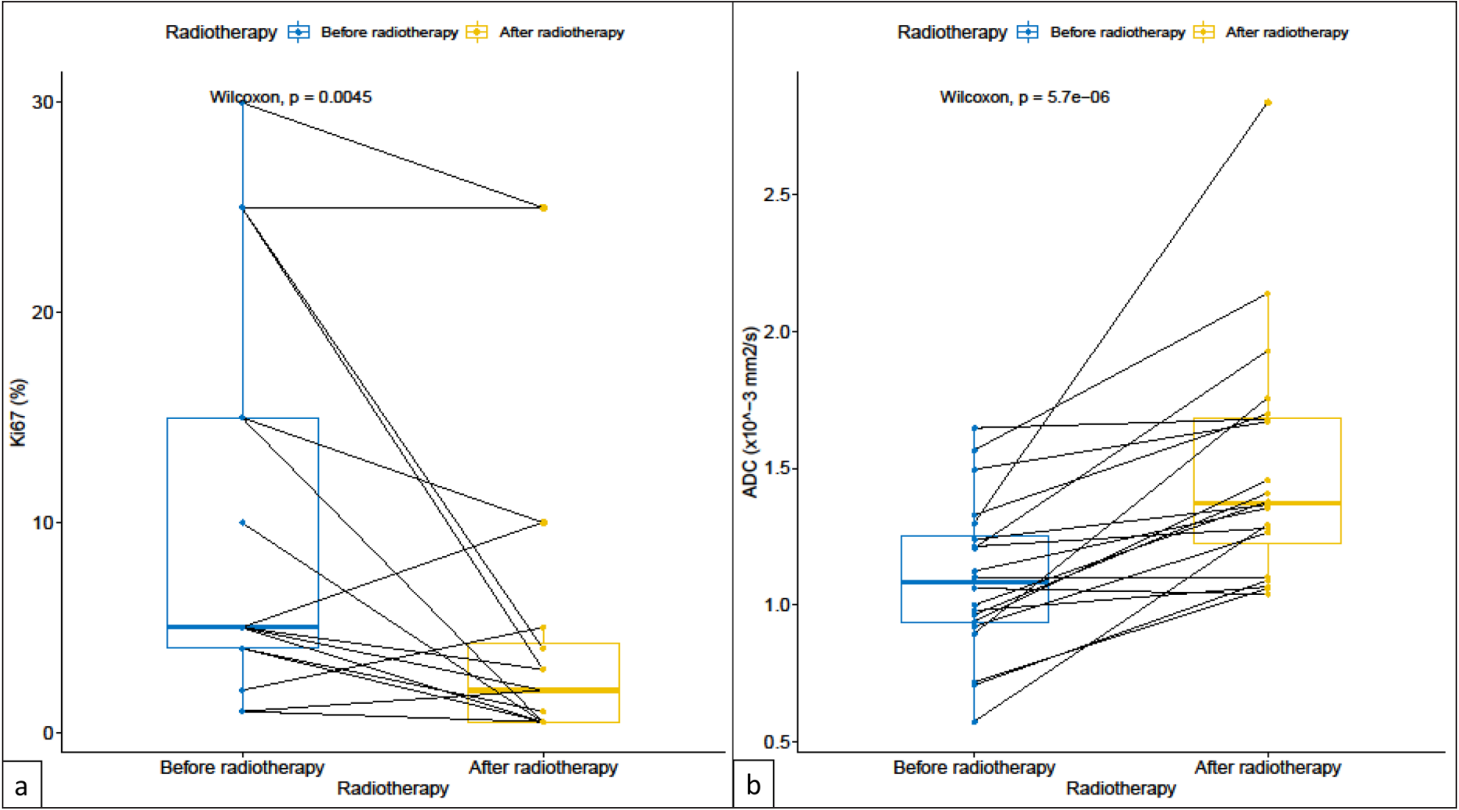
Comparison of **(a)** the variations of the Ki-67 index on tumor biopsy and surgical specimen versus **(b)** the variations of the ROI-ADC before and after neoadjuvant radiotherapy.

**Figure 3:**
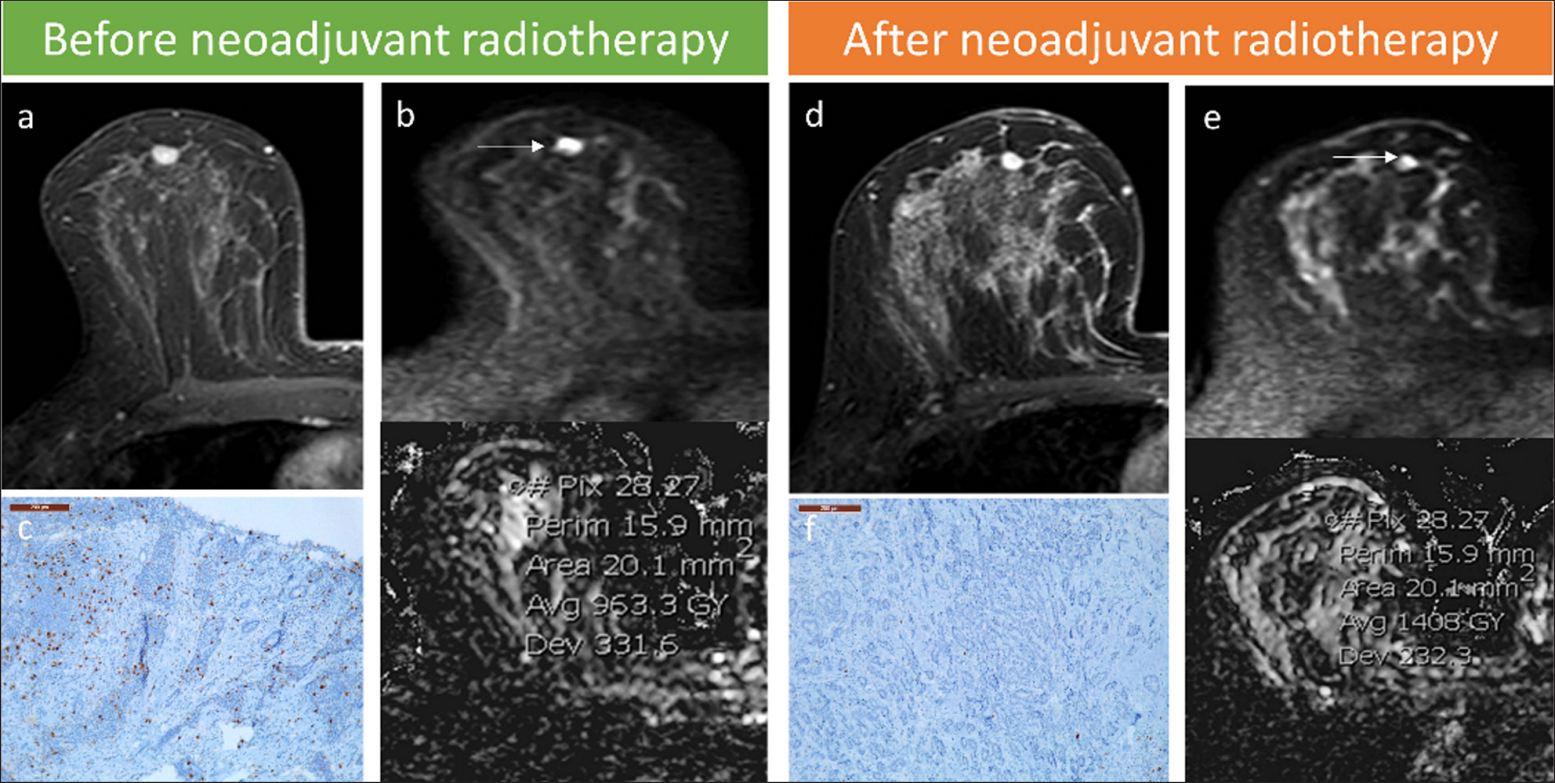
Images in a premenopausal 48-year-old woman with a right BCa. In pretherapeutic breast MRI, axial DCE T1-weighted image (WI) shows a unifocal juxta-centimetric mass **(a)**, axial DWI shows a high signal intensity mass at *b* = 800 and a ROI-ADC value of 0.963 10^-3^ mm^2^/s on ADC-map **(b)**. Immunohistochemistry shows a Ki-67 index of 25% on the core biopsy before radiotherapy **(c)**. After preoperative radiotherapy, axial DCE T1-WI **(d)** shows a stable enhancing mass. DWI shows a decrease in signal intensity of the mass on *b*=800 and a significant increase in the ROI-ADC value to 1.408 10^-3^ mm^2^/s **(e)**. Immunohistochemistry shows a Ki-67 index of less than 5% after radiotherapy on the surgical specimen **(f)**.

The mean LD on DCE sequences was 10.7 ± 4.7 mm before radiotherapy and 10.2 ± 4.6 mm (p-value = 0.033) after radiotherapy (Table 1), thus decreased by 4.7%. There was no significant change in enhancing curves. The mean ROI-ADC value was 1.093 ± 0.278 10^-3^ mm^2^/s before radiotherapy and 1.490 ± 0.429 10^-3^ mm^2^/s (p-value < 0.001) after radiotherapy. The mean LD was 8.7 ± 5.6 mm for the surgical specimen. The Ki-67 index was 9.2 ± 9.1 for the percutaneous biopsy before radiotherapy and 4.9 ± 7.5 (p-value = 0.005) after radiotherapy for the surgical specimen. After preoperative radiotherapy, a 46.7% decrease in Ki-67 index and a 36.3% increase in ADC were observed (Figure 2). Figure 3 illustrates the case of significant increase in ROI-ADC and decrease in Ki-67 index in a premenopausal 48-year-old woman with a right BCa after radiotherapy.

**Table 1:** Tumor characteristics on histology and MRI before and after radiotherapy.


			BEFORE RADIOTHERAPY N = 22	AFTER RADIOTHERAPY N = 22	P-VALUE

**Histology**	**SBR**	**1**	13	12	>0.99

		**2**	9	9	>0.99

		**NA**	0	1	–

	**Ki67, %**		9.2 ± 9.1	4.9 ± 7.5	0.005

	**LD, mm**		–	8.7 ± 5.6	–

**MRI**	**LD, mm**		10.7 ± 4.7	10.2 ± 4.6	0.03

	**TIC**	**1**	3	3	>0.99

		**2**	8	12	0.13

		**3**	10	6	0.13

	**ROI ADC, × 10^–3^mm^2^/s**		1.093 ± 0.278	1.490 ± 0.429	<0.001


Abbreviations: SBR: Scarff-Bloom-Richardson grade; Ki-67: Ki-67 index; LD: longest diameter; ROI: region of interest; ADC: apparent diffusion coefficient; TIC: time intensity curve; NA: not available.

## Discussion

Neoadjuvant preoperative radiotherapy for low-risk BCa is a new approach under study in our institution. We decided to investigate DWI in early response to radiotherapy in comparison with histopathology, including the Ki-67 index. In the present study, a 46.7% decrease in the Ki-67 index and a 36.3% increase in ADC were observed after five days of preoperative radiotherapy in 22 early BCa. Both markers may reflect tumor response to preoperative radiotherapy. However, there is no comparative study in the literature on tumor response evaluation after neoadjuvant radiotherapy in BCa.

The Ki-67 proliferation index measures the percentage of tumor cells that are positive for Ki-67 staining: the more positive cells there are, the more quickly they are dividing and forming new cells, thus reflecting the aggressiveness of the tumor. In BCa, an index of more than 20% is considered high. Ki-67 is a prognostic marker according to BCa molecular subtype and is increasingly used to assess and manage BCa [19,20]. In fact, change in Ki-67 index due to neoadjuvant chemotherapy can independently predict prognosis of patients with BCa [21,22].

In 2019, Luo and colleagues suggested that comparison of pre- and post-neoadjuvant chemotherapy ADC values can be used to estimate the change in Ki-67 index in a study including 87 patients with invasive BCa and showed a negative correlation between change in ADC values and in Ki-67 index due to neoadjuvant chemotherapy [23]. The authors suggested that changes in ADC values might be used as a surrogate marker for changes in Ki-67 index in neoadjuvant chemotherapy response of patients with invasive BCa.

In 2017, a meta-analysis demonstrated that DWI has a high sensitivity in predicting response to neoadjuvant chemotherapy [14]. The promising role of ADC values in predicting tumor response in BCa treated with neoadjuvant chemotherapy has been reported by many studies [7,11,13,14,15]. Recently, Hottat and colleagues demonstrated that a significant increase in BCa ROI ADC at DWI predicts complete pathologic and radiologic responses after one cycle of neoadjuvant chemotherapy in a cohort of 48 patients with 50 BCa [24]. Despite the lack of standardization in specific diffusion response criteria and by analogy with other functional imaging response criteria (PERCIST and EORTC), an ADC increase of 25–30% might reflect a significant response [25]. Interestingly, we observed a 36.3% increase in ADC after five days of preoperative radiotherapy, potentially illustrating a decrease in tumor aggressiveness. Therefore, ADC might be considered as an early predictor of response after neoadjuvant radiotherapy.

The limitations of this study are the small number of enrolled patients and the ADC measurements realized by only one radiologist. These preliminary results need to be confirmed in a larger population of patients.

The present study has several strengths. All patients underwent two consecutive MRI examinations before and after preoperative radiotherapy. All MRI examinations were standardized and performed prospectively using the same 3-T magnet. Post-processing was also standardized. Finally, all the patients had the same immunohistochemical tumoral profile (HR-positive/HER2 negative), which represents a homogeneous series of early-stage BCa.

## Conclusion

In conclusion, the results of this study showed a significant increase in breast tumor ROI-ADC at DWI and a significant decrease in Ki-67 index after a short neoadjuvant preoperative radiotherapy, suggesting early tumor response. Further studies with larger cohorts of patients should be realized to confirm our results.

## Data Accessibility Statements

The data that support the findings of this study are available from the corresponding author upon reasonable request.

## Additional File

The additional file for this article can be found as follows:

10.5334/jbsr.2815.s1 Supplemental Tables. Tables 1 to 2.
