# Supplementary material for: Diffusion-Weighted MRI in the Evaluation of Early-Stage Breast Cancer Treated with a Short Preoperative Radiotherapy: Preliminary Results
Source: J Belg Soc Radiol. 2023 Feb 7;107(1):8. doi: 10.5334/jbsr.2815 (PMC9912849; doi:10.5334/jbsr.2815)
Supplement: Supplemental Tables. — Tables 1 to 2. [file jbsr-107-1-2815-s1.pdf]

Supplemental table 1: 3-T MRI acquisition parameters.

**Weighting**	**T2**	**T1**	**Diffusion**	**Dyn 3D T1 fs**
**Sequence name**	TSE	TSE	DWI	eTHRIVE
**Acq. Nr of slices**	60	60	70	80
**Acq. Slice thickness/gap (mm)**	3/0	3/0	3/0	4/0
**Field of View (Read x Phase)**	320x339	320 x 339	280x378	320 x 320
**Acq. Matrix (Read x Phase)**	320x279	400x330	128 x 172	292x292
**Phase encoding direction**	LR	LR	LR	LR
**SENSE factor**	2	2	4	2x1
**Phase oversampling (mm)**	-	-	2 x 62	-
**TR (msec)/TE (msec)**	5000/60	400/10	12576/74.4	4.6/2.2
**Turbo factor**	14	5	59	40
**Flip angle**	90	90	90	10
**Fat suppression**	SPAIR	-	SPAIR	SPAIR
**b-values (s/mm^2)**	-	-	0, 50, 400, 800	-
**Acquisition duration (min:sec)**	02:45	03:27	05:39	00:52 x 8

Abbreviations: Acq: acquisition; Nr: number; TR: repetition time; TE: echo time; T2: T2-weighted; T1: T1-weighted; Dyn 3D T1 fs: dynamic three-dimensional T1-weighted with fat suppression; DWI: diffusion-weighted imaging; LR: left-right; SENSE: sensitivity encoding; SPAIR: spectral attenuation inversion recovery.

Supplemental table 2: Tumor description in the study population.

Case	Tumor type on biopsy (Luminal type)	Tumor type on specimen histology (Luminal type)	TNM
**1**	ILC (A)	ILC (A)	ypT1cN0Mx
**2**	IDC (A)	IDC (A)	ypT1bN0Mx
**3**	IDC (B)	IDC (A)	ypT1cN1aMx
**4**	IDC (A)	IDC (A)	ypT1aN0Mx
**5**	IDC (A)	IDC (A)	ypT1bN0Mx
**6**	IDC (B)	IDC (B)	ypT1cN0Mx
**7**	ILC (A)	ILC (A)	ypT1aN0Mx
**8**	IDC (A)	IDC (A)	ypT1cN1aMx
**9**	IDC (A)	IDC (A)	ypT1aN0Mx
**10**	IDC (A)	DCIS	ypTisN0Mx
**11**	IDC (A)	IDC (A)	ypT2N0Mx
**12**	IDC (A)	NA	ypT0N0Mx
**13**	IDC (B)	IDC (B)	ypT1cN0Mx
**14**	ILC (A)	ILC (A)	ypT1cN0Mx
**15**	IDC (A)	IDC (A)	ypT1bN0Mx
**16**	IDC (A)	IDC (A)	ypT1bN0Mx
**17**	IDC (A)	IDC (A)	ypT1cN0Mx
**18**	IDC (B)	IDC (A)	ypT1aN0Mx
**19**	IDC (A)	IDC (A)	ypT1bN0Mx
**20**	IDC (A)	IDC (A)	ypT1cN0Mx
**21**	ILC (A)	ILC (A)	ypT1bN0Mx
**22**	IDC (A)	IDC (A)	ypT1aN0Mx

Abbreviations: ILC invasive lobular carcinoma; IDC: invasive ductal carcinoma; DCIS: ductal carcinoma in situ; NA: not available.
